# Prevention and Treatment of Monkeypox: A Systematic Review of Preclinical Studies

**DOI:** 10.3390/v14112496

**Published:** 2022-11-11

**Authors:** Nurizzati Sudarmaji, Nurolaini Kifli, Andi Hermansyah, Siang Fei Yeoh, Bey-Hing Goh, Long Chiau Ming

**Affiliations:** 1 PAPRSB Institute of Health Sciences, Universiti Brunei Darussalam, Gadong BE1410, Brunei; 2 Department of Pharmacy Practice, Faculty of Pharmacy, Universitas Airlangga, Surabaya 60115, Indonesia; 3 Department of Pharmacy, National University Hospital, Singapore 119074, Singapore; 4 Biofunctional Molecule Exploratory Research Group (BMEX), School of Pharmacy, Monash University Malaysia, Bandar Sunway 47500, Malaysia; 5 College of Pharmaceutical Sciences, Zhejiang University, Hangzhou 310058, China

**Keywords:** vaccine, disease transmission, vaccination, outbreak, communicable disease, emerging infectious disease

## Abstract

The outbreak of monkeypox, coupled with the onslaught of the COVID-19 pandemic is a critical communicable disease. This study aimed to systematically identify and review research done on preclinical studies focusing on the potential monkeypox treatment and immunization. The presented juxtaposition of efficacy of potential treatments and vaccination that had been tested in preclinical trials could serve as a useful primer of monkeypox virus. The literature identified using key terms such as monkeypox virus or management or vaccine stringed using Boolean operators was systematically reviewed. Pubmed, SCOPUS, Cochrane, and preprint databases were used, and screening was performed in accordance with PRISMA guidelines. A total of 467 results from registered databases and 116 from grey literature databases were screened. Of these results, 72 studies from registered databases and three grey literature studies underwent full-text screening for eligibility. In this systematic review, a total of 27 articles were eligible according to the inclusion criteria and were used. Tecovirimat, known as TPOXX or ST-246, is an antiviral drug indicated for smallpox infection whereas brincidofovir inhibits the viral DNA polymerase after incorporation into viral DNA. The ability of tecovirimat in providing protection to poxvirus-challenged animals from death had been demonstrated in a number of animal studies. Non-inferior with regard to immunogenicity was reported for the live smallpox/monkeypox vaccine compared with a single dose of a licensed live smallpox vaccine. The trial involving the live vaccine showed a geometric mean titre of vaccinia-neutralizing antibodies post two weeks of the second dose of the live smallpox/monkeypox vaccine. Of note, up to the third generation of smallpox vaccines—particularly JYNNEOS and Lc16m8—have been developed as preventive measures for MPXV infection and these vaccines had been demonstrated to have improved safety compared to the earlier generations.

## 1. Introduction

The United States government has declared the monkeypox outbreak a public health emergency on 5 August 2022 following a spike in cases. This ensues declaration on 23 July 2022 by the World Health Organization (WHO) of a highest emergency alert following a worldwide surge in cases. The monkeypox virus (MPXV) originated from *orthopoxvirus* genus, *chordopoxvirinae* subfamily, and *poxviridae* family [1]. The *poxviridae* family is often brick-shaped and consists of linear double-stranded DNA and has a lipoprotein envelope. The average size of an MPXV is 200–250 nm, which is considered relatively large [2,3]. Other viruses that belong in the same genus as MPXV include variola, cowpox, camelpox, and vaccinia viruses. Variola virus (VARV) causes smallpox disease which mirrors the clinical features of MPXV—typically headache, fatigue, rash, fever, and lesions which first appeared as macular, then developed into popular, then vesicular as well as pustular. The number of lesions experienced varies from one patient to another, ranging from a few to thousands per patient, and the development of lesions into pustular is linked to the severity of the disease. The only feature that distinguishes MPXV from smallpox is the presence of lymphadenopathy in patients infected with MPXV. The lymph nodes observed in patients with MPXV can be seen as enlarged, firm, and occasionally painful. 

Complications were noted especially in unvaccinated patients and the conditions included secondary lung infection, bronchopneumonia, severe dehydration resulting from diarrhoea or vomiting, encephalitis, septicaemia and ocular infections [4,5]. The transmission of MPXV is believed to occur via skin-to-skin or face-to-face contact as well as in contact with infected objects such as clothes and beddings. Once infected, MPXV will undergo an incubation period lasting from 7 to 14 days. During this period, the virus replicates at the site of inoculation and begins spreading to the lymph nodes [6,7].

MPXV was first officially documented in an animal facility in Copenhagen, Denmark in 1958 when monkeys (*Macaca fascicularis* and *Macaca Mulatta*) supplied for polio vaccine research fell sick [8]. The first human case of Monkeypox infection was found in a child in the Democratic Republic of Congo in 1970. This child was first suspected to have smallpox [9]. From 1970 onwards, the cases continued to rise, and the infection was first spread to other African countries including Central African Republic, Cameroon, Cote D’Ivoire, Liberia Nigeria, Gabon, South Sudan, and Sierra Leone. From 1970 to 2020, nearly 29,000 suspected cases were recorded and there were over 1300 confirmed, probable, and/or possible cases of monkeypox. The countries that were most affected by this infection are the Democratic Republic of Congo, Nigeria, Congo, and Central African Republic [10]. The first case outside African countries occurred in the Midwestern United States because of the importation of infected Gambian giant rats from Ghana. The virus then spread to co-housed prairie dogs that eventually spread to humans via close contact. About 53 cases were recorded during this time in the United States [11]. Another infection case recorded in Israel after a man travelled from Nigeria and went back to Israel in 2018 [12]. One similar monkeypox case occurred in Singapore in 2019. In this case, the man had also just travelled back from Nigeria [13]. 

From 1 January 2022 until 26 October 2022, approximately 77,000 laboratory confirmed cases of monkeypox were confirmed. Statistics show the highest number of monkeypox as 28,000, which were reported in the United States of America. This is followed by approximately 9000 cases in Brazil, 7317 cases in the United Kingdom (UK), 3662 cases in Germany and 3298 cases in Colombia [14].

As the specific treatment for monkeypox is still lacking, the rising outbreak of monkeypox has raised the concerns of many especially when the COVID-19 outbreak is still going on. Therefore, we conducted this systematic review of the preclinical studies focusing on the potential monkeypox treatment and prevention, particularly vaccines. We analysed the efficacy of potential treatments and vaccination that had been tested in preclinical trials in terms of providing protection from mortality, reducing severity of clinical symptoms, reducing the viral loads and increasing antibodies to fight against MPXV. 

## 2. Methods

This review was conducted according to the Preferred Reporting Items for Systematic Reviews and Meta-Analyses (PRISMA) guidelines. The databases used for searching articles included Pubmed, Scopus and Cochrane, Medrxiv and Biorxiv and grey literature. 

In Pubmed, the following search term was used; (monkey pox OR monkeypox OR “monkey?pox” OR/Abstract]). The filter feature was used to limit the number of results. The searches in Pubmed were limited to animals and English studies only. In Scopus, the search term used was (TITLE-ABS-KEY (“monkey pox”) OR TITLE-ABS-KEY (“monkeypox”) OR TITLE-ABS-KEY (“monkey?pox”)) AND (TITLE-ABS-KEY (Therapeutics OR prevention OR control OR vaccine OR treatment OR management)). The filter was also used and the results were limited to articles only. The search term “monkey pox” OR monkeypox OR “monkeypox virus” in Title Abstract Keyword AND Therapeutics OR prevention OR control OR vaccine OR treatment OR management in Title Abstract keyword was used in the Cochrane database. In both MedRxiv and Biorxiv, the following search terms were used; monkeypox and monkey pox. 

The results from these databases were imported to Endnote X9 (Philadelphia, PA, United States) and duplicates were removed using the same app. Two investigators then independently screened the identified studies. Following removal of duplication, the remaining results were screened twice. The first screening was screening of titles and abstracts. The screening was performed based on the following exclusion criteria: (1) any articles that have no relation to monkeypox, (2) non-English studies, (3) studies that focus on other poxviruses, (4) reviews, and (5) any studies that are not accessible. Once the results were narrowed down, full-text screening was performed. Additional exclusion criteria were added during the second screening; any studies that provide lack of information or data of interest, which may also indicate high risk of bias, were removed. Data extraction of the studies was also done by all investigators independently.

With the remaining studies, they were summarised in tables according to types of treatments and vaccinations. The headings included in the table are types of samples (the types of animals or cells), intervention used with its dose and regimen, the comparison treatment or drug, method, and outcomes, which include the variables being examined.

## 3. Results

### Study Selection

**Figure 1:**
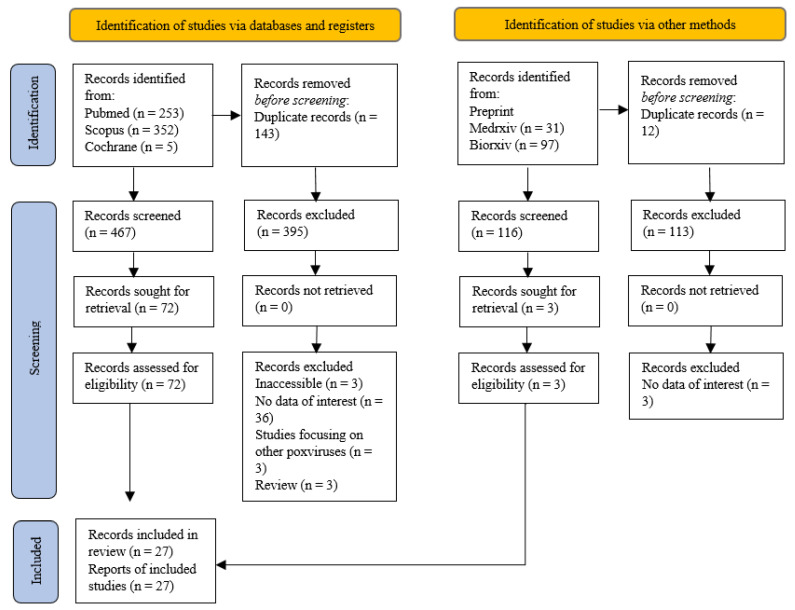
PRISMA flow chart.

The number of results produced from the mentioned search terms for each database were recorded in the PRISMA flowchart (Figure 1). A total of 467 results from registered databases and 116 from grey literature databases were screened. Of these results, 72 studies from registered databases and 3 grey literature studies underwent full-text screening for eligibility. In this systematic review, a total of 27 articles were eligible according to the inclusion criteria and were used. None of the studies from grey literature databases were eligible and a few eligible studies from the Cochrane databases were duplicates with those from Pubmed and Scopus. 

The table of evidence was prepared according to types of treatments: Tecovirimat (Table 1 and Table 2), Brincidofovir (Table 3 and Table 4), vaccines—IMVAMUNE, Vaccinia virus-immunoglobulin (VIG), ACAM2000, LC16m8, recombinant bovine herpesvirus 4 (BoHV-4), smallpox vaccine, DNA/HIV vaccines and Modified Vaccine Ankara (MVA) (Table 5 and Table 6), combination of treatments—cidofovir and Dryvax (Table 7 and Table 8) and other potential therapeutic agents (Table 9 and Table 10). The information included in the tables involve the type of samples, intervention used, comparison treatment, method and outcome measures and the corresponding findings.

## 4. Discussion

### 4.1. Efficacy and Safety of Treatments and Vaccines

#### 4.1.1. Tecovirimat

Tecovirimat is an antiviral drug, which also goes by the branded name TPOXX and code name ST-246. As previously mentioned, tecovirimat was first FDA-approved for the treatment of smallpox in 2018 but had been recently approved by the European Medicines Agency as treatment of monkeypox disease [6,42]. This antiviral drug was firstly identified in 2002 through a high-throughput screening and has been shown to have efficacy against several other orthopoxviruses besides variola and MPXV, namely cowpox, rabbitpox, ectromelia and vaccinia virus [16,43,44]. Tecovirimat acts by inhibiting VP37 protein. All members of the orthopoxvirus genus are believed to encode this protein. Inhibition of this protein will prevent VP37 from interacting with GTPase and TIP47 which consequently blocks the necessary enveloped virions from being formed [45,46]. The efficacy of tecovirimat against MPXV had been shown in several preclinical studies, including seven animal studies and one in-vitro study, as summarised in Table 1. In the reported animal studies, tecovirimat had been demonstrated to reduce the mortality of MPXV-challenged animals with at least a 90% survival rate [15,16,17,22]. However, the efficacy in preventing mortality was seen to decrease in animals with delayed treatment post-challenge [22]. A similar pattern for clinical symptoms and viral loads was seen in the studies. Untreated animals were more likely to exhibit higher viral loads and more severe clinical symptoms including lethargy, lack of appetite, respiratory distress, lesions, high temperature, weight loss, nasal discharge and respiratory failure and as for treated animals, the animals experienced mild symptoms and lower viral loads [15,17,18,20,21]. In the Huggins et al study, the tecovirimat-treated animals experienced no lesions at all. Animals receiving earlier treatment post-infection had been shown to manifest milder symptoms compared to animals receiving delayed treatment [15]. The minimum effective dose for reducing viral loads and lesions was recorded at 10 mg/kg in monkeys which is comparable to 400 mg of tecovirimat in humans [16,20].

#### 4.1.2. Brincidofovir

Hexadecyloxypropyl-cidofovir (HDP-CDV) or CMX001, famously known as Brincidofovir (BCV), is an alkoxyalkyl lipid ester conjugate of cidofovir (CDV). Post intravenous administration, only a very small amount of the drug reaches the kidney as it not largely taken up by transporters, hence, limiting the risk of nephrotoxicity unlike CDV [47,48]. BCV has an effect against double-stranded DNA viruses and exerts its antiviral effect by penetrating the infected cells upon administration. The drug will then be cleaved to CDV and phosphorylated to form cidofovir diphosphate, an active metabolite. This metabolite, in turn, prevents DNA polymerization by competing with deoxycytosine-5-triphosphate (dCTP) for viral DNA polymerase. This eventually disrupts viral replication [49]. BCV was first indicated for smallpox treatment in both paediatrics and adults with a dosage of 200 mg once weekly for 2 doses for those with 48 kg or above. However, it has now been under consideration to use against monkeypox infection [50,51,52]. BCV had also been shown to have positive outcomes in previous animal studies testing against several poxviruses [53,54,55]. In an MPXV-challenged animal study, the plasma concentration of BCV was analysed at different doses; 5 and 20 mg/kg and was tested with single and repeated administration. The plasma concentration was found to fall below the limit of quantification (BLQ) after 24 h (5 mg/kg dose) and 36 h (20 mg/kg) for single dose. Similar results were recorded for multiple doses of 5 mg/kg, but the concentration fell below BLQ by 48 h for dose of 20 mg/kg. In the same study, the efficacy of this drug was assessed by observing the mortality rate and clinical signs of MPXV-challenged black-tailed prairie dogs. A trend can be observed on the survival rate. Animals administered BCV were shown to have a delay in mortality in comparison to animals receiving treatment on the day of challenge and one day post-challenge. However, the highest rate of survival achieved was only 57%, which suggests a lack of efficacy of BCV in the treatment of MPXV. Despite the low efficacy, Hutson CL et al. suggested that BCV may be effective if given in combination with another drug such as tecovirimat [23].

#### 4.1.3. Monkeypox Virus Vaccines

Dryvax, one of the first-generation smallpox vaccines, was made by replicating the vaccinia virus. This vaccine was shown to have promising results against this virus and was used to eradicate smallpox [56,57]. In the Zielenski Rj et al. study using the cynomolgus macaques model, the vaccine used involved the Wyeth strain. The vaccine was integrated with varying interleukins, IL-2 and IL-15, but Wyeth/IL-2 vaccines were served as the comparison group together with the Modified Vaccinia Ankara (MVA)-vaccinated group. In the study, Wyeth/IL-2 and MVA-vaccinated groups exhibited milder clinical symptoms compared to the Wyeth/IL-15 group. The Wyeth/IL-15 group had also shown to have a delay in healing. The vaccinia plaque reduction neutralizing antibody titres (PRNT 80%) were observed in the same study and MVA-treated animals were shown to have 4-fold compared to the other groups at 6 weeks post-vaccination, which implies poor efficacy of integrated Wyeth strains smallpox vaccines [29]. In other animal studies by Buchman et al., the Dryvax vaccine was used as a comparison group to a smallpox vaccine with A33, B5, L, A27 (ABL) and aluminium hydroxide (ABLA). The outcome of the study indicated favourable results of Dryvax against MPXV by which the mortality rate was seen to be reduced to zero and significantly lower viral loads in comparison to the negative control group (*p* < 0.05) [30]. Despite the efficacy, this vaccine was found to be linked with severe, rare side effects including Stevens-Johnson syndrome, myocarditis, pericarditis, eczema vaccinatum, encephalitis, progressive vaccinia and occasionally death. Hence, this encouraged the development of smallpox second- and third-generation vaccines [58].

ACAM2000, is live vaccinia virus vaccine derived from cell cultures in Vero Cells and is considered as a second-generation smallpox vaccine. On 31 August 2007, ACAM2000 was licensed by the FDA for individuals with high risk of smallpox infection [59]. The efficacy of this vaccine in overcoming the high mortality rate and infection severity had been demonstrated in several animal studies involving prairie dogs and cynomolgus monkeys [25,28,31]. In one of the studies, there was no reported significant change in blood or chemistry parameters within the first 15 days after vaccination. This was consistent with the pathological symptoms observed in the immunised models, which was in contrast with the control group. Apart from that, the mortality rate was seen to be reduced with further delays in receiving vaccination post-challenge, but no statistically significant survival benefit was calculated. This study used a single dose of 1 × 10^5^ PFU of ACAM2000 [31]. In another study, the survival rate of models reached 100% and low levels of viremia was detected with the booster dose. However, some clinical symptoms were present including lesions. Based on the results, it was suggested that the prime-boost approach may be useful in obtaining an optimal effect of the vaccine as a high level of antibodies were detected following the booster dose [28]. This vaccine is said to have a similar profile as Dryvax and the efficacy is as good as the first-generation. However, there are still reported adverse effects which may induce the risk of developing complications in vaccinated individuals [31,58,60,61]. 

Examples of third-generation smallpox vaccines are IMVAMUNE and LC16m8 vaccines. IMVAMUNE also goes by different marketing names; Modified Vaccinia Ankara-Bavarian Nordic (MVA-BN; Germany), JYNNEOS (the United States) and IMVANEX (the European Union). Unlike the first- and second-generation smallpox vaccines, IMVAMUNE had no reported complications linked with the first-generation vaccines. Hence, it is currently developed as an effective and safe vaccine in the prevention of smallpox and other poxviruses. However, higher doses may be required for IMVAMUNE [28,30]. This vaccine was manufactured based on a highly attenuated strain of vaccinia virus—MVA virus—which underwent multiple changes including mutations and deletions to lose its capacity to replicate efficiently in most mammalian cells and humans in order to be used in the IMVAMUNE vaccine [38]. The efficacy of this vaccine against MPXV had been tested in a number of animal studies [25,28]. One of the studies demonstrated that the prairie dog models that had been immunised with IMVAMUNE one day post-infection had shown to have reduced weight loss by Day 16 post-challenge in comparison to unvaccinated group, but no statistically significant difference was calculated (*p* > 0.05). Though, animals that had been administered with IMVAMUNE three days post-challenge recorded greater weight loss than the control group by Day 16 post-infection, which questions the vaccine’s ability in preventing mortality in delayed immunisation. As for comparing lesion counts, the lesion counts in one-day post-challenge IMVAMUNE-vaccinated animals were found to be significantly lower than the control group (*p* < 0.05) [25]. In a different study, all but 2 vaccinated cynomolgus macaques survived by day 11 post-infection. All immunised animals were seen to have increased in body weight by day 14 post-challenge. Milder clinical symptoms such as depression and dyspnea were observed in dead vaccinated animals compared to the control group. However, other surviving animals exhibited little to no clinical signs [28]. In a 1970s study in Germany, MVA vaccines were tested on approximately 120,000 people together with the Lister vaccine and there were no serious adverse reactions reported [62]. 

The LC16m8 vaccine, a highly attenuated vaccine and another example of third-generation smallpox vaccines, was initially developed using the Lister strain of smallpox vaccine in rabbit kidney cells under low temperature conditions in Japan in the 1970s [63]. The efficacy of this vaccine in providing protection and reducing the infection severity in animal models had been demonstrated. In the studies, no animals succumbed to infection and had shown little to no clinical signs of MPXV infection. Any signs developed were found the be milder than the control groups [26,64]. In the Iizuka et al., study, they signified that LC16m8 has the capability to provide long-term immunity against the MPXV virus in Macaca fascicularis species. However, the duration of the immunity was still unclear. Hence, further investigations are required to determine the duration [26].

#### 4.1.4. Other Potential Therapeutic Agents

In the Mucker et al. study in 2018, they evaluated the use of monoclonal antibodies in the prophylaxis of severe MPXV infection. The particular antibodies used were 7D11 and c8A, which were produced by BioFactura [40]. These antibodies target the mature virion (by C7D11) and extracellular virion (by c8A), and eventually inhibit further action of the virions [65]. The study demonstrated that these antibodies are effective in providing protection and reducing the signs and symptoms of the disease. A total of 2 out of 3 treated animals survived and exhibited no symptoms, and it was found that C7D11 was capable of decreasing the viral load by ~90% with high dose ->1250 PFU/mL [40]. However, since the sample for this study is very small (n = 3), there is less reliability on the results and different outcomes may be projected with bigger samples. It is also noteworthy that this is the first study to use marmosets as the model for the MPXV study, hence requiring further varying studies to evaluate the use of monoclonal antibodies as prophylactic treatment for MPXV in determining the doses and immunologic responses induced by these antibodies.

Interferon-Beta (IFN-β) was another potential agent and FDA approved this drug for multiple sclerosis. IFN-β acts by stimulating the production of IFN-stimulated genes. This stimulation of these genes will activate apoptosis, allowing the active action of macrophages and natural killer cells to inhibit the synthesis of proteins. The major histocompatibility complex-1/II expression on the surface of antigen presenting cells will also be upregulated because of these genes [66]. This agent was tested by Johnston et al., 2012. In their in-vitro study, IFN-β was assessed for its ability to inhibit the production and spread of MPXV in monolayers of HeLa cells and normal derma fibroblasts. For the outcomes, it was shown that 2000 U/mL of IFN-β was capable of inhibiting the spread of MPXV for a minimum 91% in all cells. This efficacy is thought to be caused by one molecule, MxA, which was found to have an antiviral activity against a number of RNA viruses such as influenza and measles viruses. However, the exact mode of action of MxA against MPXV is unclear and requires further studies [41].

### 4.2. Limitations of Reported Studies

#### 4.2.1. Animal Models

The ideal characteristics of an animal model to be used in preclinical studies should be those that are similar in a number of traits; possess similar clinical features, disease course, and mortality as humans, capable of mimicking similar transmission of the pathogen as to humans, and having a similar dosage of drugs to produce similar effects to that in humans. In addition, a model is considered ideal if a large number of animals can be provided in the research. However, varying animal models were used in the reported studies in the tables above with monkeys being the most common one used. The distinct traits or characteristics of different animals such as pharmacokinetics or histopathological changes may have contributed to varying outcomes of the studies [67]. Hence, it is unfair to compare two studies with different animals used despite involving the same poxvirus. In the studies above, the list of animal models includes monkeys (*Macaca fascicularis*, *Macaca Mulatta*, crab-eating macaques, Rhesus macaques, and marmosets), prairie dogs, ground squirrels, and mice.

Among the animals mentioned above, humans share the most similar physiology with monkeys. These animals exhibit an identical duration of onset and clinical features of disease as humans when challenged with MPXV via IV or aerosol route. Hence, a number of parameters can be used as a reference or comparison i.e., temperature, vital signs etc. However, there are a few limitations that should be noted. Monkey models require a high dose of virus to develop a symptom in comparison to other models (10^6^~10^7^ PFU of MPXV for monkeys). Although these models can be inoculated via aerosol or the intratracheal route, they still do not entirely mimic all the natural transmission routes of infection in humans [68,69]. The types of monkey models used in studies should also be taken under consideration as only certain species are vulnerable to MPX infection and varying species may have a different disease onset and severity. For instance, the outbreak in the US in 1959 was first spread by *Macaca fascicularis* and *Macaca mulatta*. These animals were co-housed with another monkey species, African *Chlorocebus Aethiops*, who surprisingly did not exhibit any of the MPXV symptoms at all [70]. This explains that the species factor could have contributed to varying outcomes in studies. Eric et al. (2018) also outlined the possibility of the effect of gender on the disease’s severity and symptoms as they observed that female marmosets had fewer viremia and oral shedding and developed higher number of lesions compared to male marmosets [40]. Thus, it is rather challenging to correlate the efficacy and safety of drugs in the models to in humans. 

Rodents require a dose of virus that is far lower than the amount needed in monkey models, ranging from 12,000~32,000 PFU of MPXV. The inoculation routes for MPXV challenge for these small animals were through intranasal, intraperitoneal or cutaneous, which slightly but not entirely mimic the transmission of virus as in humans [22,71,72,73]. However, ground squirrels and prairie dogs may not be the ideal models for these studies as the availability of these animals is rather restricted due to a low reproductivity rate. Even if they are available, they are likely to be captured from the wild. Hence, there is a possibility of these animals being exposed to other pathogens and unknown external or internal factors which could have led to the misinterpretation of the outcomes [69]. Furthermore, ground squirrel models experienced only a few similar symptoms as the ones developed in humans. Thus, limited parameters can be used as reference. In addition to that, a limited species of mice can be used as a model in MPXV studies as a number of certain strains of these rodents were not vulnerable to this infection. Immunocompromised mice developed symptoms when exposed to MPXV but this model does not mimic the natural infection of MPXV. STAT1^(−)^ mice, however, were found to have high sensitivity to this infection. Therefore, they were used in studies testing drug efficacy [74,75].

#### 4.2.2. Monkeypox Virus Strains

Based on the severity of disease and geographical origin, MPXV strains were categorised into two clades; Congo Basin (CB) and West African (WA) MPXV [76]. It is important to note that the severity of disease that a strain caused may greatly affect the outcome of the studies. Both strains resulted in different disease severity and CB had been linked to higher severity. In the Hutson et al. (2009) study, C57BL/6 and BALB/c mice were challenged intranasally (IN) or subcutaneously in the footpad (FP) with 10^5^ PFU of either WA or CB MPXV strain. CB MPXV-challenged mice FP developed oedema on day 6 p.i. on BALB/c and C57BL/6 mice, but greater oedema was identified in BALB/c mice. Severe swellings were noticed in two BALB/c and one C57BL/6 mice in the following day and the oedema resolved by day 13 p.i. Throughout the study, only one BALB/c mice had weight loss, as much as 7.3% of its initial mass. As for WA MPXV-challenged FP mice, mild oedema developed on day 7 p.i. This oedema was less severe in comparison to CB MPXV FP mice and the oedema completely resolved by day 9 or 11 p.i. Unlike CB MPXV FP animals, none of the WA MPXV FP mice lost any weight. CB MPXV IN-challenged animals. Ruffled fur was observed in four BALB/c mice and weight loss was noted in most of the inoculated animals, ranging between 3 to 19% of initial weight. Other than that, no other signs developed. In contrast, no observable morbidity signs were seen in WA MPXV IN animals [77]. In a different study with similar aims, prairie dogs were used as models. In this study, 10^4^ PFU of either WA or CB MPXV strains were introduced to the animals via intradermal via scarification (ID) or IN. Animals challenged with CB MPXV ID or IN had a recorded rise in temperature, which was calculated as significantly higher than WA MPXV-challenged animals. Lesions started to appear on CB-MPXV animals by day 6 p.i. and on WA-MPXV animals by day 6–9 p.i. By day 15 p.i., about 3 CB MPXV prairie dogs succumbed to infection (two ID and one IN) and none for WA MPXV prairie dogs. The DNA of MPXV was also detected in swabs in the range of day 6–21 p.i. for MPXV ID prairie dogs, and on day 3–21 and day 6–18 p.i. for WA MPXV IN and CB MPXV in prairie dogs, respectively [71]. According to these studies, it is evident that CB MPXV strain is more virulent in comparison to the WA strain as animals tend to have a higher mortality rate and severity of clinical manifestations of this disease. The variability of these strains may have given rise to a distinct impact on the end results of these studies.

### 4.3. Clinicians and Researchers Notes

In terms of treatment of monkeypox, no antiviral treatment specifically for MPXV is available yet. However, tecovirimat, known as TPOXX or ST-246, is an antiviral drug indicated for smallpox infection and had been approved by the European Medicines Agency for MPXV infection in January 2022. Apart from tecovirimat, an anti-smallpox drug brincidofovir, also known as CMX001 or Tembexa, is also under the consideration for use as treatment for monkeypox infection [51,52]. Tecovirimat inhibits a specific protein in orthopoxviruses, namely p37, which is an essential protein for producing virions of poxviruses [78,79]. On the other hand, brincidofovir inhibits the viral DNA polymerase after incorporation into viral DNA [80]. The ability of tecovirimat in providing protection to poxvirus-challenged animals from death had been demonstrated in a number of animal studies [44,81]. Brincidofovir had also been shown to be effective against orthopoxviruses [46]. However, the issue that comes with these drugs is that they are yet to be approved by the Food and Drugs Administration (FDA) specifically for the treatment of monkeypox. We also included the limitation of the reported studies in Table 1, Table 2, Table 3, Table 4, Table 5, Table 6, Table 7 and Table 8 which MPXV researchers could pinpoint the appropriate animal model and virus strain to be used as reference for their future research.

With the resurgence of MPXV infection and a lack of efficacious and safe drugs currently available to tackle the infection, developing treatments and preventive measures for this disease has become a critical aspect to look further into. From this review, we have learned that up to the third generation of smallpox vaccines, particularly JYNNEOS and Lc16m8, have been developed as preventive measures for MPXV infection. These vaccines had been demonstrated to have improved safety compared to the earlier generations of smallpox vaccines, which had been reported to cause complications in those receiving these vaccines. Clinicians need to be precautious as the vaccines may have less efficacy under special circumstances. In this case, more focus should be directed in developing anti-viral drugs to manage and control MPXV infection, especially in those in high-risk groups, particularly those who are under immunocompromised condition.

#### Strength and Limitation of this Review

Due to the lack of feasibility of clinical trials and the unethical nature of introducing MPXV to human subjects, efficacy is based on animal models which we have included all relevant preclinical studies related to treatment and prevention of monkeypox. 

Although this review is inclusive with all preclinical studies from the early 2000s, it is important to note that older studies may have used old preclinical guidelines, thus, not consistent with the current guidelines for preclinical studies.

## 5. Conclusions

To date, at the global scene, no approved treatment or vaccine for monkeypox is available. While the effectiveness of repurposed drugs in treating MPXV among human has not been evaluated, potential treatment benefit based on preclinical studies including animal efficacy data could be particularly useful. Third-generation smallpox vaccines, particularly JYNNEOS and Lc16m8, have been developed as preventive measures for MPXV infection and these vaccines had been demonstrated to have improved safety compared to the earlier generations of smallpox vaccines. Furthermore, tecovirimat has been shown to be effective against various orthopoxviruses in multiple animal challenge models. The limitations of the reported studies, particularly from the aspects of animal models and MPXV strains indicated that monkeys may be the ideal model for testing safety and efficacy of drugs for MPXV infection. These models not only reflect similar routes of transmissions as in humans but also identical duration of course and clinical manifestations. As for the MPXV strain, using the WA MPXV strain may not entirely represent the real-life condition as it is less virulent hence this strain is less likely to cause a wide spread of infection. Moreover, the severity it caused is milder. Hence, the CB MPXV strain would be preferred as it may help in providing the optimal outcomes of drugs. 

## Figures and Tables

**Table 1:** Characteristics of Tecovirimat (or ST-246) tested in preclinical studies.

Ref.	Samples	Intervention	Comparison	Method		
Russo, et al. [15]	Cynomolgus Macaques (n = 62, 31 M & 31 F, aged 2–7 years)	10 mg/kg of tecovirimat on day 1, 2, 3, 4, 5, 6, 7 or 8.	Placebo.	Animals were administered with 1.0 × 105 PFU MPXV (MPXV strain Zaire-V79-I-005) following anesthetised with 2–6 mg/kg of Telazol. Animals were distributed into 11 groups, each receiving different treatment regimens; (1) Placebo A (2) treatment on day 1 (3) treatment on day 2 (4) treatment on day 3 (5) treatment on day 4-A (6) treatment on day 4-B (7) treatment on day 5 (8) treatment on day 6 (9) treatment on day 7 (10) treatment on day 8. All administered with 10 mg/kg tecovirimat except for placebo groups which receive vehicle delivery, followed by 0.3 ± 0.03 mg/kg of meloxicam. The monkeys were monitored for clinical signs, lesions, weight loss and survival.		
Grosenbach, et al. [16]	Crab-eating macaques (n = 20)	0.3, 1, 3, 10 & 20 mg/kg of tecovirimat on day 4 post-challenge	Placebo	Macaques challenged with 5 × 107 PFU of monkeypox virus (Zaire 1979 strain) via IV inoculation on Day 0 and administered with tecovirimat on or after day 4 following challenge. Varying doses of 0 (placebo), 0.3, 1, 3, and 10 mg/kg were evaluated in the first study and the pharmacokinetic of tecovirimat were evaluated in the second study using doses of 0 (placebo), 3, 10, and 20 mg/kg.		
**Ref.**	**Samples**	**Intervention**	**Comparison**	**Method**		
Berhanu, et al. [17]	Cynomolgus macaques (n = 32, 16 F & 16 M)	10 mg/kg of Tecovirimat for 14 days	Combination of tecovirimat and ACAM2000^®^	32 macaques were grouped randomly into 4 groups of 8 macaques. At day 0, ~5 × 10^7^ PFU of MPXV Zaire-79 were introduced to animals and they were mock-vaccinated or vaccinated with 2.5 μL/2.5 × 10^5^ to 12.5 × 10^5^ PFU of ACAM2000^®^ at day 3 followed by 14 days of oral placebo or 10 mg/kg of tecovirimat. At day 63, surviving animals then rechallenged with ~5 × 10^7^ PFU of MPXV Zaire-79 to determine the protective immunity. Throughout the study, animals were monitored for temperature, body weight, lesion counts and any clinical symptoms.		
Smith, et al. [18]	Prairie dogs (n = 18, aged 3, weighed 876–976 g)	30 mg/kg daily tecovirimat for 14 days	Placebo (Vehicle-treated)	Others were challenged with 10^5^ PFU of MPXV strain ROC-2003-358 and distributed into 4 groups of 4 (2 M & 2 F); 3 groups treated with ST-246 either at day 0 (prophylactically), day 3 (post-infection) or during post-rash onset (therapeutically) for 14 days. On day 3, one group received vehicle. 2 extra females remain uninfected and received ST-246 on the same day.		
Smith, et al. [19]	African green monkey kidney cells (BSC-40 cells)	5, 1.5, 0.5, 0.15, 0.05, 0.015, 0.005, and 0.0015 µM of ST-246	Untreated.	CPE assay	Plaque size evaluation & IHC	Comet reduction assay & IHC
				Cells seeded & grown in 96-well plate. Cells infected at 0.1 MOI & incubated for 1 h in 5% CO_2_ at 35 °C. Viral inocula then replaced with growth media containing ST-246 (5, 1.5, 0.5, 0.15, 0.05, 0.015, 0.005, and 0.0015 µM) and 2% FBS & incubated for 3 days in 5% CO_2_ at 35.5 °C. Cells then stained with 2× crystal violet and their absorbance were measured at 570 nm.	Cells seeded and grown in 6-well plate. 25 PFU of virus added to each well and incubated for 1 h in 5% CO_2_ at 35.5 °C. Cells then washed, added with RPMI containing ST-246 (0.5, 0.15, 0.05, 0.015, and 0.005 µM) added to well & incubated for 3 days in 5% CO_2_ at 35.5 °C. Cells then fixed with 10% formalin. Immunohisto-chemistry (IHC) performed.	Similar procedures as plaque size evaluation. During the final incubation, plates were placed at ~5° angle.
Jordan, et al. [20]	cynomolgus monkeys, *Macaca fascicularis* (n = 15 for dose determining study and n = 20 for PK analysis study)	(1) Dose determining study: 3, 10, 30, and 100 mg/kg daily PO of ST-246 for 14 days (2) PK analysis study: 10, 20 & 30 mg/kg of ST-246–14 days & multiple doses for 10 & 30 mg/kg and single-dose for 20 mg/kg.	Vehicle	Determination of dose	PK of ST-246	
				Animals challenged with 5 × 10^7^ PFU of MPX strain Zaire 79 and randomly divided into 5 groups of 3, receiving vehicle or varying doses of ST-246 3 days post-challenge; (1) Control (Vehicle) (2) 3 mg/kg ST-246 (3) 10 mg/kg ST-246 (4) 30 mg/kg ST-246 (5) 100 mg/kg ST-246	Animals were given primate biscuit slurry & divided into 3 groups receiving different doses of ST-246 by oral gavage; (1) 10 mg/kg for 14 days (n = 6, 3 F & 3 M) (2) 20 mg/kg (n = 8, 4 F & 4 M–single dose) (3) 30 mg/kg for 14 days (n = 6, 3 F & 3 M)	
Huggins, et al. [21]	Cynomolgus monkeys (n = 8 M, mean age 6.2 ± 0.7 years and mean weight 6.2 ± 1.3 kg)	300 mg/kg/day of ST-246 for 14 days at day 0 or 3 post-challenge.	Vehicle	Animals challenged with 5 × 10^7^ PFU of MPX strain Zaire 79 & randomly distributed into 2 groups of 4. For each group, 3 animals randomly chosen & administered with ST-246 and 1 animal received vehicle; Group 1—received at day 0 Group 2—received at day 3 post-challenge		
Sbrana, et al. [22]	13-lined ground squirrels, *Spermophilus tridecemlineatus* (n = 6 for PK Analysis & n = 59 for experimental infection & dose regimens)	(1) PK analysis study: 100 mg/kg of ST-246 (2) Experimental infection & dose regimens: 100 mg/kg PO of ST-246 at 0, 24, 48, 72 & 96 h post-challenge for 14 days	Placebo (HPMC + Tween 80 without drug)	PK analysis study	Experimental infection & dose regimens	
				6 squirrels were bled & given one dose of ST-246. Animals were bled at 1, 2-, 4-, 8- & 24-h post-treatment & blood sample collected.	59 squirrels randomly grouped into 7 groups; (1) Placebo + 100 PFU of MPX-ZAI-1979-005 virus strain (n = 9) (2) Normal group–uninfected (n = 7) (3) ST-246-treated at 0 h post-challenge (n = 8) (4) ST-246-treated at 24 h post-challenge (n = 9) (5) ST-246-treated at 48 h post-challenge (n = 9) (6) ST-246-treated at 72 h post-challenge (n = 9) (7) ST-246-treated at 96 h post-challenge (n = 8)	

**Table 2:** Characteristics of Tecovirimat (or ST-246) tested in preclinical studies (Outcome measures).

Ref.	Outcome Measures	Findings
Russo, Grosenbach, Brasel, Baker, Cawthon, Reynolds, Bailey, Kuehl, Sugita, Agans and Hruby [15]	Mortality	49 out of 54 animals receiving tecovirimat and 2 of 7 placebo animals survived. All animals receiving tecovirimat on day 1–5 and 7 survived. As for animals receiving tecovirimat on day 6 and 8 recorded 66% and 50% survival rate, respectively. 2 animals in day 8 group were moribund therefore were eliminated.
	Weight loss	Treated animals: had shown to have increase in body weight. Placebo group: experienced weight loss on day 5 onward and only plateaued after day 38. The weight loss in groups receiving treatment day 1–4 was seen to decrease after challenge was calculated as significant but not for groups receiving treatment after day 4. All groups then continued experiencing weight loss until at least day 8.
	Clinical signs	Placebo animals: Clinical scores increased in placebo animals with peak between day 5 and 19 following challenge. The progression of disease increased the severity of symptoms and slow recovery rate was observed. The clinical signs observed were respiratory distress, dyspnea, diaphragmatic breathing, increased respiratory rate, respiratory failure, no appetite, no or scant stool, less urination and lethargy. Animals receiving tecovirimat at day 1 and 2: had lower clinical scores groups receiving tecovirimat on day 3 or 4: had higher score. The peak reached at day 8.
Grosenbach, Honeychurch, Rose, Chinsangaram, Frimm, Maiti, Lovejoy, Meara, Long and Hruby [16]	Survival rate	Only 1 of 20 untreated macaque (5%) survived in the first study and about 95% of treated macaque survived. Minimum effective dose was 3–10 mg/kg. These doses had also demonstrated to reduce viral loads and lesion counts especially with 10 mg/kg. In treatment-delay study: animal receiving treatment at day 4 or 5 following challenge had higher survival rate (83%) than those receiving at day 6 (50%). In treatment-duration study: animals treated with 3 daily doses of 10 mg/kg of tecovirimat at day 4 post-exposure had 50% survival rate. 100% survival rate achieved with 5 and 7 daily treatments and 80% survival calculate for 10 daily treatments.
	Pharmacokinetic analysis	In the evaluation of pharmacokinetic study, the following results were recorded after 14th dose; minimum concentration (Cmin) of 169 ng/mL, maximum concentration (Cmax) of 1444 ng/mL and mean concentration (Cavg) of 598 ng/mL.
Berhanu, Prigge, Silvera, Honeychurch, Hruby and Grosenbach [17]	Survival rate Clinical symptoms	All ACAM2000^®^/tecovirimat-treated animals survived whereas other groups succumbed to infection at day 7–12 post-infection. Tecovirimat-treated animals showed reduced clinical symptoms with normal temperature maintained whereas signs were severe, and temperatures declined dramatically in mock/placebo-treated & ACAM2000^®^/placebo-treated animals.
	Clinical symptoms	Tecovirimat-treated animals showed reduced clinical symptoms with normal temperature maintained whereas signs were severe, and temperatures declined dramatically in mock/placebo-treated & ACAM2000^®^/placebo-treated animals.
	Viral load	Tecovirimat-treated group had also reduced viral loads in blood (*p* < 0.0001) and lesion counts (*p* < 0.01) compared to placebo-treated groups. Though no significant differences calculated between placebo-treated groups or between tecovirimat-treated groups (*p* > 0.05).
Smith, Self, Weiss, Carroll, Braden, Regnery, Davidson, Jordan, Hruby and Damon [18]	MPXV DNA	Vehicle-treated animals: On day 4–6, viral DNA was identified in blood & oral swabs. ST-246-treated animals (day 0): Only detected in oral swabs on day 14 but no longer detected from day 26. ST-246-treated group (day 3): Detected in oral swabs of on day 8 and peaked between day 8–12. Viral DNA in blood was undetected. Animals treated with ST-246 after rash onset: Detected in varying days in 3 animals (day 4, 6 or 12 before rash onset).
	Signs	Vehicle-treated animals: Manifested lack of appetite, swollen face, nasal discharge & congestion on day 8 post-infection. Weight loss observed on day 6–8. 3 of 4 succumbed to infection on day 10–12. Surviving animal manifested only red, puffy nose on day 8–9. ST-246-treated animals on day 0 and day 3 post-infection: All survived and remained clinically normal for 30 days of observations. Weigh loss did not exceed 5%. Animals treated with ST-246 after rash onset: Manifested signs similar to vehicle-treated animals on day 8 post challenge but less severe. 3 animals had rash consisting of 5–50 lesions on day 10 on chest, abdomen & back
Smith, Olson, Karem, Jordan, Hruby and Damon [19]	CPE assay	5 MPXV strains were used to measure the level of protection of cells monolayer. Protection was detected at conc. Of 5–0.05 µM. The absorbance slightly decreased at 5 µM due to the 9ompound’s cytotoxicity. EC_50_s of ST-246 for MPXV strains ranged 0.023 µM ± 0.0026 to 0.039 µM ± 0.0016.
	Plaque size evaluation & HIC	Reduction in plaque size observed at 0.5, 0.15, and 0.05 µM. The reduction observed at 0.015 µM was ~50% and the plaque size at 0.005 µM was similar to untreated wells.
	Comet reduction assay & IHC	Inhibition of comet tail formation was observed at 0.5–0.05 µM. Minimal inhibition observed at 0.015 µM and none at 0.005 µM.
Jordan, Goff, Frimm, Corrado, Hensley, Byrd, Mucker, Shamblin, Bolken, Wlazlowski, Johnson, Chapman, Twenhafel, Tyavanagimatt, Amantana, Chinsangaram, Hruby and Huggins [20]	Mortality, lesions & vDNA.	1 control animal died on day 11 post-challenge & 2 vehicle-treated animals became moribund & euthanised on days 13 & 14 post-challenge. All ST-246-treated animals survived. The difference in survival rate between control & treated animals was significantly different (*p* < 0.001). Lesions developed in treated animals were lower than vehicle-treated group & virus replication in treated groups were >1000x lower than control group.
	Dose determining study	Levels of blood exposure by 10 mg/kg dose was comparable to those in humans with 400-mg of ST-246.
	PK analysis study	Steady-state conc: 16–21% drug accumulation recorded by day 6. Saturable absorption: Occurred at dose of 800-mg/day Urinary excretion of ST-246: <0.03% of dose–vert low.
Huggins, Goff, Hensley, Mucker, Shamblin, Wlazlowski, Johnson, Chapman, Larsen, Twenhafel, Karem, Damon, Byrd, Bolken, Jordan and Hruby [21]	Mortality & lesions	Control animals: Exhibited ~1500 lesions & died by day 13 post-infection. ST-246-treated animals: No lesions observed on treated animals.
	Viral loads	Control animals: Viral loads exceed 10^8^ genomes/mL between days 5–8. ST-246-treated animals: ~5 logs less than control animals.
Sbrana, Jordan, Hruby, Mateo, Xiao, Siirin, Newman, Travassos Da Rosa and Tesh [22]	PK analysis study	Clinical appearance: Remained normal. Concentration of ST-246 in blood: 15,000 ng/mL at 8 h post-treatment & declined until undetectable at 24 h post-treatment.
	Experimental infection & dose regimens: Mortality	ST-246-treated animals at 0, 24, 48 & 72 h post-infection: All survived. ST-246-treated animals at 96 h post-infection: 67% survived. Placebo group: All died between days 6–9.
	Experimental infection & dose regimens: Virologic & antibodies	Normal group: No detectable viremia. Placebo group: High titre of MPXV. ST-246-treated animals at 0, 24, 48 & 72 h post-infection: No detectable viremia & antibodies. ST-246-treated animals at 96 h post-infection: Low titre viremia (0.2 & 2.1 PFU/mL) & had detectable antibodies.
	Experimental infection & dose regimens: Histopathologic & immunohisto-chemical	Normal group: MPXV-antigen negative. Clinically normal. Placebo: Enlarged spleens, liver & lung consolidation, fibrinoid necrosis in mantle & marginal zone of splenic lymphoid follicles, oedema & haemorrhage in lung. ST-246-treated animals at 0 & 24 h post-infection: No observable changes except for macrophage proliferation in spleen follicular area (in 24 h group) ST-246-treated animals at 48 h post-infection: Inflammatory infiltrates identified in lungs and small number of inflammatory foci in liver lobules. ST-246-treated animals at 72 h post-infection: Span of mantle zone of spleen reduced, apoptotic hepatocytes & inflammatory infiltrates in liver. ST-246-treated animals at 96 h post-infection: Spleen fibrinoid necrosis and severe lymphoid depletion observed.

**Table 3:** Characteristics of Brincidofovir tested in preclinical studies.

Ref.	Samples	Intervention	Comparison	Method	
Hutson, et al. [23]	Black-tailed prairie dogs, Cynomys ludovicianus Plasma conc. Of BCV study; n = 16, 8 M & 8 F, aged 2–4 years & weighed 913–1230 g Efficacy study; n = 28 F, aged—4 years	(1) Plasma conc. Of BCV study; 3-doses oral regimen; 20 mg/kg & 5 mg/kg (2) Efficacy study; 3-doses oral regimen; 20 mg/kg (1st dose) & 5 mg/kg (2nd & 3rd dose)	Vehicle	Plasma concentration of BCV following single and repeat oral administration	Determining the efficacy of BCV in animals challenged with MPXV
				Animals were distributed & received 3-doses oral regimen; (1) 20 mg/kg (n = 8) (2) 5 mg/kg (n = 8)	Animals distributed into 4 groups; (1) Positive control—Vehicle-treated (n = 7) (2) Treated with BCV on day 1 pre-infection (n = 7) (3) Treated with BCV on day 0 (n = 7) (4) Treated with BCV on day 1 post-infection (n = 7) West African MPXV strain was inoculated to animals.

**Table 4:** Characteristics of Brincidofovir tested in preclinical studies (Outcome measures).

Ref.	Outcome Measures		Findings
Hutson, Kondas, Mauldin, Doty, Grossi, Morgan, Ostergaard, Hughes, Nakazawa, Kling, Martin, Ellison, Carroll, Gallardo-Romero and Olson [23]	Plasma concentration of BCV following single and repeat oral administration	Single oral administration	Median time of Tmax fell within 4–8 h range. The plasma concentration was below limit of quantification (BLQ) by 24 h for 5 mg/kg and by 36 h for 20 mg/kg.
		Repeat oral administration	Plasma concentration for predose prior to third administration were BLQ. Tmax is between 4–6 h. The plasma concentrations were BLQ on general by 24 h for 5 mg/kg and by 48 h for 20 mg/kg.
	Determining the efficacy of BVC in animals challenged with MPXV	Clinical signs	Vehicle group: appeared at day 5 post-infection in one animal & at day 8 post-infection for others. Clinical signs vary among animals. Animals receiving BCV (n = 7): Lowest average maximum clinical score recorded Other animals: Have scores of 8.1 (those received treatment on the day of infection), 8.7 (those received BCV 1 day p.i) and 9.1 (those in vehicle group)
		Mortality	Varying survival rate calculated for different groups: 57% (those received treatment 1 day prior infection), 43% (those received treatment on day of infection), 29% (those received BCV 1 day p.i.) and 14% (vehicle group) Animals receiving BCV before challenge had shown to have delay in mortality.

**Table 5:** Characteristics of vaccines tested in preclinical studies.

Ref.	Samples	Intervention	Comparison	Method	
Parker, et al. [24]	Cast/EiJ mice (aged 6–12 weeks)	160 µg of Recombinant Vaccinia virus-immunoglobulin (rVIG) intraperitoneally (IP) at days 7 & 14 prior challenge & days 0, 4, 5 or 6 post-challenge.	Cidofovir & VIG	Mice were challenged with 2.4 × 10^4^ PFU of MPXV Zaire. Animals were treated with IP injection of rVIG at days 7 & 14 before inoculation & at days 0, 4, 5 & 6 post-infection. Mice were then bled at days 6, 8, 10 & 15 post-challenge.	
**Ref.**	**Samples**	**Intervention**	**Comparison**	**Method**	
Shannon Keckler, et al. [25]	Prairie dogs (n = 86)	(1) 500 µL of 1 × 10^8^ TCID IMVAMUNE at day 1 or 3 (2) Single dose of 1 × 10^5^ PFU of ACAM2000	Dryvax & Unvaccinated	170 × LD_50_ study	2 × LD_50_ study
				50 animals were grouped into 10 groups based on age, weight & sex; (1) Day 1—single-dose of × 105 pfu Dryvax (n = 5) (2) Day 3—single-dose of × 105 pfu Dryvax (n = 5) (3) Day 1—single-dose of × 105 pfu ACAM2000® in 10 µL PBS (n = 5) (4) Day 3—single-dose of × 105 pfu ACAM2000 in 10 µL PBS (n = 5) (5) Day 1—single-dose of 500 µL 1 × 10^8^ TCID IMVAMUNE (n = 5) (6) Day 3—single-dose of 500 µL 1 × 10^8^ TCID IMVAMUNE (n = 5) (7) Day 1 unvaccinated (n = 5) (8) Day 3 unvaccinated (n = 5) (9) Day 1 uninfected (n = 5) (10) Day 3 uninfected (n = 5) Under anesthesia with 5% isoflurane gas, 40 animals were introduced with 4.31 × 10^6^ PFU of MPXV in 10 µL PBS via nasal inoculation and remaining 10 were with 10 µL PBS only.	36 animals were distributed as follows; (1) Day 1—single-dose of × 105 pfu ACAM2000 in 10 µL PBS (n = 8) (2) Day 3—single-dose of × 105 pfu ACAM2000 in 10 µL PBS (n = 8) (3) Day 1 single-dose of 500 µL 1 × 108 TCID IMVAMUNE (n = 8) (4) Day 3 single-dose of 500 µL 1 × 108 TCID IMVAMUNE (n = 8) (5) Unvaccinated (n = 4) All animals in 2 × LD_50_ were challenged with 2.25 × 104 pfu of MPXV.
Iizuka, et al. [26]	Cynomolgus monkeys, *Macaca fascicularis* (n = 4, 13 M & 1 F, weighed 2500–4500 g.	1 × 108 PFU/mL of LC16m8 vaccine	Lister vaccine and unvaccinated group	Animals were distributed into 5 groups – one control group and 4 other groups receiving different vaccines at varying months before route10^6^ PFU of MPXV strain Zr-599 was inoculated via subcutaneous. The groups were as follows; (1) unvaccinated group (n = 4) (2) LC16m8 at 6 months pre-exposure (n = 3) (3) LC16m8 at 12 months pre-exposure (n = 3) (4) Lister at 6 months pre-exposure (n = 2) (5) Lister at 12 months pre-exposure (n = 2). Animals were observed for survival and clinical symptoms until 3 weeks at maximum. Temperature and body weight were measured afterwards, and virus isolation were measured from blood samples.	
Franceschi, et al. [27]	STAT1^(−/−)^ mice (n = 129)	Recombinant bovine herpesvirus 4 (BoHV-4) vectors [BoHV-4-A-CMV-A29LgD106ΔTK (A29L), BoHV-4A-EF1α-M1RgD106ΔTK (M1R) and BoHV-4-A-EF1α-B6RgD106ΔTK (B6R)]	Unvaccinated.	Mice were grouped into 13 cages of 5 mice and some received vaccination intraperitoneally; Cage 1 & 2—unvaccinated Cage 3—vaccinated with DMEM without PBS) Cage 4—primarily vaccinated with MVA at day 0 and vehicle booster at day 23 Cage 5—vaccinated with MVA on both day 0 and 23 Similar regimens of Cage 4 & 5 were applied to 6 & 7, 8 & 9 and 10 & 11 but with different vaccines—A29L, M1R and B6R, respectively. Cage 12 & 13 had similar regimen but were vaccinated with combination of the 3 vectors. Mice in cage 2–13 were challenged with 2 × 10^5^ PFU of MPXV at day 50.	
Hatch, et al. [28]	Cynomolgus Macaques (n = 24, 12 F & 12 M, weigh 2.5–4.5 kg, age 2–4 years)	(1) 10^8^ TCID_50_ of **Imvamune** [Single (28 days pre-challenge) and two-doses (56 days and 28 days pre-challenge)] (2) 2.5 × 10^5^ to 12.5 × 10^5^ PFU of **ACAM2000** (Single dose–28 days pre-challenge)	Placebo (TBS)	Animals distributed into 4 groups of 6; (1) Control group—inoculated with 0.5 mL of Tris-buffered Saline (TBS) 28 days pre-infection (n = 6 M) (2) Vaccinated with one-dose of ACAM2000 (n = 6 F) (3) Vaccinated with one-dose of IMVAMUNE (n = 6 M) (4) Vaccinated with 2 doses IMVAMUNE (n = 6 F). Animals were introduced to10^5^ PFU of monkeypox virus strain Zaire 79, NR-2324.	
Zielinski, et al. [29]	50 µL 1 × 10^8^ PFU of Smallpox vaccine (Wyeth strain) with integrated IL-15 (Wyeth/IL-15) intradermally	Unvaccinated, Wyeth/IL-2 and MVA vaccines (MVA, MVA/IL-2, MVA/IL-15)	20 monkeys distributed into 7 groups, receiving different vaccines; (1) Wild type Wyeth (n = 3) (2) Wyeth/IL-2 (n = 3) (3) Wyeth/IL-15 (n = 3) (4) MVA (n = 2) (5) MVA/IL-2 (n = 3) (6) MVA-IL-15 (n = 3) (7) Unvaccinated (n = 3) 5 × 10^7^ pfu of monkeypox virus (Zaire 79 strain) was introduced to animals 3 years post-vaccination.	50 µL 1 × 10^8^ PFU of Smallpox vaccine (Wyeth strain) with integrated IL-15 (Wyeth/IL-15) intradermally	
Buchman, et al. [30]	cynomolgus macaques, *Macaca fascicularis* [n = 30 (16 F & 14 M) for 3-dose study & n = 12 (7 F & 5 M) for 2-dose study)	1 mL of Protein-based smallpox vaccine (comprising vaccinia virus membrane proteins A33, B5, L1, A27 (ABL) & Aluminium hydroxide (ABLA) ± CpG–100 µg each for ABL & 20 or 100 µg each for ABLA)	Non-vaccinated (CpG/alum without proteins) & positive control (Dryvax)–vaccinated once at day 0 for each study.	3-dose study	2-dose study
				Divided into groups of 5; Vaccinated intramuscularly with vaccine containing ABL + CpG/alum or ABLA + alum or CpG/Alum at 0, 4 & 12 weeks. Monkeys challenged with 1 mL of 2 × 10^7^ PFU of MPXV 5 weeks after last vaccination.	Distributed into groups of 3; Vaccinated intramuscularly with vaccine containing ABLA + CpG/alum at 0 & 4 weeks. Monkeys challenged with 1 mL of 2 × 10^7^ PFU of MPXV 4 weeks after last vaccination.
Marriott, et al. [31]	Cynomolgus macaques (n = 24, 12 M & 12 F, aged ≥ 22 months)	4.4 × 10^8^ PFU/mL of ACAM2000	Dryvax & ACAM2000 gycerol-phenol diluent (negative control)	Animals distributed into 3 groups of 8 & received different treatments on day 0; (1) Negative control—ACAM2000 gycerol-phenol diluent (2) 1.5 × 10^8^ PFU/mL of Dryvax (3) 4.4 × 10^8^ PFU/mL of ACAM2000 Animals challenged with 0.5 mL 3.8 × 10^7^ PFU of monkeypox strain Zaire 79 61 days post-vaccination via IV inoculation.	
Nigam, et al. [32]	Chinese rhesus macaques (n = 9) & Indian rhesus macaques (n = 6)	(1) 0.6 mg of JS2, JS7 & JS8 DNA/HIV vaccines as primer on week 0 (2) 1 × 10^8^ PFU/mL of MVA/HIV 48 vaccines as booster on weeks 8 & 32	-	Macaques distributed into 3 groups of 5; (1) Vaccinated with JS2 DNA/HIV vaccine (2) Vaccinated with JS7 DNA/HIV vaccine (3) Vaccinated with JS8 DNA/HIV vaccine All were then boosted with MVA/HIV 48.	
Earl, et al. [33]	Monkeys, *Macaca Mulatta* (n = 30)	50 µL, 2 × 10^8^ infectious unit of Recombinant MVA or MVA/KB9-5 at weeks 0 & 4–IM, ID or into palatine tonsils (PT).	Non-recombinant MVA	Monkeys divided into 4 groups & control group was further subdivided into 3 groups; (1) Control group 1.1 Non-recombinant MVA via ID (n = 3) (2) 1.2 Non-recombinant MVA via IM (n = 2) (3) 1.3 Non-recombinant MVA in PT (n = 2) (4) MVA/KB9-5 via IM (n = 8) (5) MVA/KB9-5 via ID (n = 8) (6) MVA/KB9-5 in PT (n = 7) All monkeys were introduced with 20 infectious unit of SHIV/89.6P pathogen intrarectally at week 41. 6 of animals (2 from each immunized groups) challenged with 5 × 10^7^ IV infectious unit of MPXV 2.7 years later & 2 naïve monkeys added as negative controls.	
Saijo, et al. [34]	Cynomolgus monkeys, *Macaca fascicularis* (n = 15, 14 F & 1 M, aged 3–4 years, weighed 2180–3100 g)	1 × 10^8^ PFU/mL of LC16m8 vaccines	Mock-vaccinated & Lister	Monkeys were divided into 6 groups. 3 groups will be challenged with MPXV intranasally (IN) and another 3 groups subcutaneously (SUBQ); (1) Mock-vaccinated (Naïve) group–IN (n = 2) (2) Lister-vaccinated–IN (n = 3) (3) LC16m8-vaccinated–IN (n = 3) (4) Mock-vaccinated (Naïve) group–SUBQ (n = 2) (5) Lister-vaccinated–SUBQ (n = 2) (6) LC16m8-vaccinated–SUBQ (n = 3) All monkeys were challenged with 1 × 10^6^ PFU of MPXV at week 5 post-vaccination.	
Heraud, et al. [35]	Rhesus macaques, *Macaca Mutatta* (n = 14)	**Subunit recombinant vaccine** consisting of 4 plasmids (4 mg each)–administered IM (3 mg) and ID (1 mg) + **proteins** prepared in alum/mixed with CpG-B ODN 2006–administered IM	Mock-vaccinated	Monkeys were groups into 5 & challenged with 5 × 10^7^ PFU at week 35 post-vaccination for groups 1, 4 & 5 and at week 41 for group 2 & 3; (1) DNA + protein-CpG (2) Proteins-CpG (3) Proteins + Alum (4) DNA (5) Mock-vaccinated + alum + CpG	
Stittelaar, et al. [36]	cynomolgus macaques, *Macaca fascicularis* (n = 35)	**10^8^ of SUBQ MVA-BN vaccine**	Sham-vaccinated & Elstree-RIVM vaccine	Animals distributed into 5 groups; (1) Vaccinated with 10^8^ TCID_50_ MVA-BN twice SUBQ (2) Vaccinated with 2 × 10^6^ of MVA-BN + intracutaneous (IC) Elstree-RIVM vaccine 10 days later (3) Vaccinated with one dose of Elstree-RIVM IC (4) Vaccinated one dose of Elstree-RIVM (5) Sham-vaccinated	
Hooper, et al. [37]	Rhesus macaques	Smallpox DNA vaccine	Hantaan virus DNA vaccine (negative controls) & Dryvax (positive controls)	Monkeys divided into 4 groups; (1) Vaccinated with 4 pox DNA vaccine (n = 3) (2) Vaccinated with L1R DNA vaccine (n = 2) (3) Negative controls–vaccinated with Hantaan virus DNA vaccine (n = 3) (4) Positive controls–vaccinated with Dryvax (n = 2) Monkeys were challenged with 2 x 10^7^ PFU of MPOV-Z79 IV.	
Earl, et al. [38]	Cynomolgous monkeys, *Macaca fascicularis* (n = 24)	10^8^ PFU of MVA	Unvaccinated & Dryvax	Monkeys were groups into 4; (1) Vaccinated with 10^8^ PFU of MVA IM at T = 0 & 2 months later (2 dose) (2) Vaccinated with 10^8^ PFU of MVA IM at T = 0 & percutaneous injection of Dryvax 2 months later (3) Received no vaccination at T = 0 & Dryvax 2 months later Control-Unvaccinated	

**Table 6:** Characteristics of vaccines tested in preclinical studies (Outcome measures).

Ref.	Outcome Measures	Findings
Parker, D’Angelo, Buller, Smee, Lantto, Nielsen, Jensen, Prichard and George [24]	Antiviral activity	rVIG did exhibit potent antiviral activity against MPXV strain. EC50 value for MPXV was ~2× less than VIG.
	Level of rVIG	rVIG remained detected 14 days prior to challenge. Highest level recorded was at day 6 post-challenge & it decreased gradually & reached ~20 μg/mL by day 15 post-challenge.
	Mortality	rVIG-vaccinated mice: Zero mortality/rapid weight loss. Mice not vaccinated with rVIG: 80% mortality rate recorded.
Shannon Keckler, Salzer, Patel, Townsend, Akazawa, Doty, Gallardo-Romero, Satheshkumar, Carroll, Karem and Damon [25]	Mortality	PEP-vaccinated animals: Delayed mortality observed (75%) IMVAMUNE-vaccinated group: on Day 1 had the lowest mortality rate at 12% and 62% was recorded for IMVAMUNE^®^ on day 3 as the highest mortality rate. 170 × LD_50_ study: no significant survival benefit calculated as only one animal survived–one that was vaccinated with three-days post-exposure Dryvax or IMVAMUNE. 2 × LD_50_ study: 88% animals in one-day post-exposure IMVAMUNE and 50% in one-day post-exposure ACAM2000^®^ group survived. In three-days post-exposure vaccines, ACAM2000-vaccinated group recorded 62% and as for IMVAMUNE - 38% survival rate. There was no statistically significant survival benefit calculated.
	Weight loss	170 × LD_50_ study: No significant weight loss recorded. 2 × LD_50_ study: Both groups vaccinated on Day 1 and 3 experienced weight loss at day 7. ACAM2000^®^-vaccinated animals lost −5% by day 13 and IMVAMUNE^®^ lost −3% by day 16 at maximum. In comparison, unvaccinated animals reached a maximum −14% weight loss at Day 16. Despite the huge difference, no significant difference calculated using two-way ANOVA. For animals vaccinated at day 3, ACAM2000^®^-vaccinated animals lost −16% by day 16 and IMVAMUNE^®^ lost −19% by day 16 at maximum. Only ACAM2000^®^-vaccinated animals was shown to have significance difference in median weight loss compared to unvaccinated animals. The weight loss was said to be related to the mortality rate.
	Antibody titre & vDNA	Antibody titres: both 170 × LD_50_ and 2 × LD_50_ studies, the titres were below detection level on Day 0 but increased over 14 days. ACAM2000^®^ group in 2 × LD_50_ study had shown a log increase which was not seen in other groups and in other study. vDNA: the median peak in blood for animals vaccinated on Day 1 in 170 × LD_50_ study was 10^5^–10^7^. Only Dryvax^®^ had statistically significant lower median peak in comparison to unvaccinated group. In oral swabs, the median peak vDNA was similar to in blood but only ACAM2000^®^ was shown to have significantly lower median peak. In 2 × LD_50_ group, the vDNA was much lower (10^4^–10^6^ in blood and 10^4^–10^7^ in oral swabs) and only IMVAMUNE^®^ had significant lower median peak vDNA compared to unvaccinated group.
	Lesion counts	170 × LD_50_ study: no significant differences shown for ACAM2000^®^ or IMVAMUNE^®^ vaccinated animals. 2 × LD_50_ study: animals vaccinated at day 1 had fewer secondary lesions compared to unvaccinated animals and for IMVAMUNE^®^ group, the lesion counts were significantly lower using two-way ANOVA (*p* < 0.05). For animals vaccinated at Day 3, ACAM2000^®^ group had lower lesion counts but the result was not statistically significant by two-way ANOVA.
Iizuka, Ami, Suzaki, Nagata, Fukushi, Ogata, Morikawa, Hasegawa, Mizuguchi, Kurane and Saijo [26]	Survival rate	Unvaccinated monkeys: Only one survived. Vaccinated group: All monkeys survived.
	Clinical symptoms	Unvaccinated monkeys: All were tested positive for MPXV isolation from buffy coat fractions and manifested varying clinical symptoms–weight loss of ~10%, loss of appetite, decreased activity, papulovesicles, ulcer and diarrhea. The lesions appeared in this group varied from 95 to over 1000 in number. Vaccinated group: only 2 of 3 monkeys vaccinated at 6 months before challenge tested positive for MPXV isolation. Other groups were all tested negative. The number of plaques formed on these 2 positive-tested monkeys ranged 1–5 plagues in one monkey (animal 4638) and 6–20 in another monkey (animal (4636). No lesions observed in vaccinated monkeys’ organs except for LC16m8-6M and LC16m8-12M groups (at site of challenge). Vaccinated animals maintained body weight but no significant difference observed between LC16m8vaccinated and the Lister-vaccinated monkeys.
	Antibodies	Unvaccinated monkeys: negative for IgG antibody in response to vaccinia virus antigens. Vaccinated group: During observation period, OD_405_ values for Lister group remained plateau and the values decreased for LC16m8 group. The values differences were statistically significant. Immediate antibody responses observed in vaccinated groups within 7-days post-exposure.
Franceschi, Parker, Jacca, Crump, Doronin, Hembrador, Pompilio, Tebaldi, Estep, Wong, Buller and Donofrio [27]	Survival rate	Mice in cage 4, 5 and 9 were 100% protected and 1 mouse in cage 13 succumbed to infection.
	Weight loss	In terms of weight loss, cage 4 lost ~5% or less on day 6–8 post-infection, cages 10, 11, 12 and 13 lost 15%, 15%, 11%, and 15% of initial weight, respectively on day 8 (calculated as significant).
Hatch, Graham, Bewley, Tree, Dennis, Taylor, Funnell, Bate, Steeds, Tipton, Bean, Hudson, Atkinson, McLuckie, Charlwood, Roberts and Vipond [28]	Humoral immune responses	Increased neutralising antibodies detected 6 days pre-challenge. First-dose of IMVAMUNE (13 U/mL of max. median titre) is significantly lower (*p* < 0.01) than ACAM2000 group (132 U/mL) but not the booster IMVAMUNE-vaccinated group (69 U/mL). IgG antibodies increased in ACAM2000 group (2.4 log10 AIU/mL, 9 days pre-challenge) and 2nd-dose of IMVAMUNE group (2.3 log10 AIU/mL, 7 days pre-challenge). 9 days post-challenge, antibodies in all vaccinated groups peaked. Booster IMVAMUNE group had significantly higher antibodies (*p* < 0.05) than ACAM2000 group (4.0 and 3.5 log10 AIU/mL, respectively) & remained higher at day 14 & 21 post-infection (*p* < 0.05).
	Cell-mediated responses	Pre-challenge: Increased lymphocytes in control & ACAM2000 group. CD3^+^, CD4^+^ and CD8^+^ increased slightly with first dose of IMVAMUNE but following 2nd dose, no further rise detected. Post-challenge: Increased immune cells in control group and a significant difference (*p* < 0.05) between control & 2 doses of IMVAMUNE group observed 9 days post-challenge (increased NK cells in control group). B cells & CD8^+^ cells were significantly higher (*p* < 0.05) in surviving one-dose IMVAMUNE-vaccinated animals (n = 4) than ACAM2000 group and two-dose IMVAMUNE group. 6 days post-challenge: IFN-γ and IL-6 increased in control group and one-dose IMVAMUNE group but no increased IFN-γ detected in ACAM2000 and two-doses IMVAMUNE group.
	Clinical signs & mortality	Weight loss: 10–18% in control group & less severe in other groups. All-vaccinated groups had increased body weight 14 days post-infection. Mortality: All control animals (n = 6) and 2 of one-dose IMVAMUNE animal died on day 7–11 post-challenge. Signs appeared 5 days post-infection onwards. Control animals manifested progressing depression, dyspnea, and nasal discharge. One-dose IMVAMUNE-vaccinated dead animals experienced mild depression and dyspnea 6 days pos-infection onwards before death. Surviving animals were clinically normal except for lesions. Lesions: Appeared 6 days post-infection. Greatest number of lesions recorded in control group (51/animal) and fewer in vaccinated groups.
Zielinski, Smedley, Perera, Silvera, Waldmann, Capala and Perera [29]	Post-vaccination lesions	Wyeth & Wyeth/IL-15 groups: Erythematous at site of inoculation 48 h post-vaccination & progressed into vesicular lesions by day 4. 3 animals vaccinated with Wyeth/IL-15 had healed lesions 30 days post-vaccination. Wyeth/IL-2 group: Milder lesions. MVA & integrated MVA groups: induration & erythema at site of inoculation within 24 h post-vaccination but no vesicle formation & healed within few days.
	Vaccinia plaque reduction neutralizing antibody titres (PRNT 80%)	6 weeks post-vaccination: MVA & integrated MVA had 4× higher than Wyeth & integrated Wyeth groups, but integrated MVA had no measurable impact on antibody titre. Integrated Wyeth had 2× increased in antibody titre compared to wild type Wyeth. 3 years post-vaccination: 14 animals had no vaccinia & monkeypox neutralizing antibodies detected at lowest serum dilution (1:10). Only 3 animals had detectable antibody (Titres of 50 in 1 Wyeth/IL-15-vaccinated monkey, 25 in 1 MVA/IL-2 and 25 in 1 MVA-IL-15 monkey)
	Post-challenge clinical observations	Temperatures: Only 2 animals (both unvaccinated) had temperatures greater than 103 °F at day 3 post-challenge. Weight: 1 MVA/IL-2-vaccinated monkey lost weight more than 4% by day 6 post-challenge. 1 unvaccinated gained weight during euthanasia. Another 6 animals had weight loss at day 12 onwards.
Buchman, Cohen, Xiao, Richardson-Harman, Silvera, DeTolla, Davis, Eisenberg, Cohen and Isaacs [30]	Mortality	All animals vaccinated with Dryvax survived with little-to-no clinical symptoms. All animals vaccinated with 3-dose vaccines containing CpG/alum survived. Only 4 of 5 animals receiving ABLA/alum survived.
	Viral Loads	Viral loads for Dryvax-vaccinated animals remained unmeasurable except for one animal (~2 × 104 genome copies/mL 3 days post-challenge). Viral loads in Dryvax-vaccinated animals were significantly lower than negative control group (*p* < 0.05). Viral loads in ABLA/Cpg/alum-vaccinated animals were significantly lower than control group 3, 5 & 12 days post-infection (*p* < 0.05).
Marriott, Parkinson, Morefield, Davenport, Nichols and Monath [31]	Protective efficacy	ACAM2000-vaccinated animals: All survived. Had high level of neutralizing antibodies, ranged 12,047–88,037. Dryvax-vaccinated animals: Had high level of neutralizing antibodies post-infection, ranged 33,483–74,688.
	Viremia	ACAM2000-vaccinated animals: No observable virus in blood sample. 3 animals had very low but detectable level of virus in throat swabs. Dryvax-vaccinated animals: No observable virus in blood sample & throat swabs. Control: Viral replication was apparent.
	Clinical signs	ACAM2000-vaccinated animals: Little-to-no observable clinical symptoms. One animal experienced rash-like skin eruptions at challenge site 5–7 days post-challenge & healed within 2 days. Dryvax-vaccinated animals: 3 animals experienced rash-like skin eruptions at challenge site 5–7 days post-challenge & healed within 2 days. Control group: Experienced severe signs post-infection; lack of appetite, lethargy.
Nigam, Earl, Americo, Sharma, Wyatt, Edghill-Spano, Chennareddi, Silvera, Moss, Robinson and Amara [32]	CD4 & CD8 T cell responses	Not detected until 1st booster. CD8 detected in 4 of 5 macaques on one-week post-booster vaccination and in 12 of 15 macaques by week 8. CD4 & CD8 detected after 2nd booster. CD8 was 10–15× higher than CD4 on week 1 post-2nd booster. CD4 detected in 11 of 15 animals (0.02–0.14% of CD4) & CD8 was detected in all animals (0.06–2.1& of CD8).
	Neutralizing antibody	Remained undetected until 2nd booster. Antibody titre expanded to 285–9615 at 3 weeks after 2nd booster & 2× higher at week 8.
Earl, Americo, Wyatt, Anne Eller, Montefiori, Byrum, Piatak, Lifson, Rao Amara, Robinson, Huggins and Moss [33]	Antibody titres	VACV MV binding antibody titres reduced to <1:6400 & neutralizing titres to <1:563.
	Peak viral load	Vaccinated group had 2.5 logs lower peak viral load than control group. The difference between the vaccinated & control animals was significantly different (*p* < 0.0054)
	Lesions	Control group: Huge number of lesions & experienced serious clinical symptoms. Vaccinated animals: Few to no lesions & no clinical signs appeared.
Saijo, Ami, Suzaki, Nagata, Iwata, Hasegawa, Ogata, Fukushi, Mizutani, Sata, Kurata, Kurane and Morikawa [34]	Protection & clinical symptoms	Naïve—IN: Loss ~10% body weight. Symptoms experienced at day 10 post-challenge included lack of appetite, diarrhea, skin rash, rhinorrhea, reduced activity & conjunctival discharge. Lister & LC16m8-vaccinated—IN: All survived & manifested no symptoms & weight loss. Naive—SUBQ: Loss ~15% body weight. Animals showed severe symptoms including lesions. Lister-vaccinated–SUBQ: Clinically normal. LC16m8: Clinically normal except for local lesions at site of challenge.
	Viremia, IgG & neutralizing antibody responses	Viremia was observed in Naïve—IN group & not in Lister—IN & LC16m8—IN. Viremia was detectable in Naïve—SUBQ, LC16m8—SUBQ & one Lister—SUBQ animal. Viremia was highest in Naïve–SUBQ. VV antigen-specific IgG was detected in Lister & LC16m8-vaccinated monkeys by 2 weeks post-vaccination.
	Skin lesions	LC16m8-vaccinated: Pustules, scabs & scarring induced. The max. size of lesion (at 2 weeks post-vaccination) was 27 ± 11 mm^2^. Lister-vaccinated animals: Pustules, scabs & scarrings induced. The max. size of lesion (at 2 weeks post-vaccination) was 115 ± 65 mm^2^. The lesions were more granulomatous than LC16m8-vaccinated animals.
Heraud, Edghill-Smith, Ayala, Kalisz, Parrino, Kalyanaraman, Manischewitz, King, Hryniewicz, Trindade, Hassett, Tsai, Venzon, Nalca, Vaccari, Silvera, Bray, Graham, Golding, Hooper and Franchini [35]	Antibody titre	DNA + proteins group: High levels of antibody titres than proteins alone & DNA alone group.
	Neutralising antibody	DNA only group: No neutralizing Abs detected in serum. Other groups: Detected in serum.
	Lesions	DNA + protein group: Had mild symptoms—<25 lesions. Papules did not develop into vesicles & healed within few days. Proteins-CpG & Proteins + alum: 2 animals had mild symptoms, 3 had 25–99 lesions (moderate) and 2 had 100–200 lesions (severe). DNA only group: Innumerable lesions & developed into pustules. Animals were euthanised on days 11, 17 & 21 post-challenge.
Stittelaar, Van Amerongen, Kondova, Kuiken, Van Lavieren, Pistoor, Niesters, Van Doornum, Van Der Zeijst, Mateo, Chaplin and Osterhaus [36]	Cell-mediated immune responses	T cells secreting IFN-γ were detected in all vaccinated groups but declined over 5 weeks later & became undetectable at 15 weeks. Lymphoproliferation responses were observed in all vaccinated groups.
	Humoral immune responses	Group vaccinated with MVA-BN twice: IgG antibodies increased rapidly after 1st vaccine & booster after 2nd vaccine. High level of neutralising antibody detected in all animals one week after 2nd vaccine. Group vaccinated with MVA-BN + Elstree-RIVM: IgG antibodies detected by 2 weeks post-MVA-BN vaccination and boosted after Elstree-RIVM vaccination. Neutralising antibody only detected after Elstree-RIVM vaccination. Groups vaccinated with Elstree-RIVM (group 3 & 4): High IgG detected by 2 weeks post-vaccination & peaked within week 4.
Hooper, Thompson, Wilhelmsen, Zimmerman, Ait Ichou, Steffen, Schmaljohn, Schmaljohn and Jahrling [37]	Neutralising antibodies	Animals vaccinated with 4pox & L1R: Became detectable after vaccination. Negative control: Remained undetectable until day 10 post-challenge & titre remained below <40.
	Immunogen-specific antibodies	4pox-vaccinated: Antibodies specific to all A27L, L1R, B5R & A33R VACV antigens only detectable in one animal. Another 2 only had measurable antibodies specific to 3 antigens (A27L, B5R & A33R) L1R-vaccinated: Antibodies specific to L1R were relatively high. Dryvax-vaccinated: One animal had no detectable antibodies & one animal had antibodies specific to all 4 antigens.
	Mortality & Clinical signs	Negative control: Developed severe disease & died on day 7, 10 & 14. Dead animals showed presence of vesiculopustular rash, lymphadenopathy, splenomegaly, pulmonary oedema, necrosis of bone marrow & haemorrhage in heart, urinary bladder, digestive tract, uterus & lymph nodes. Animals also experienced fever & elevated white blood cells. Dryvax-vaccinated: Clinically normal. L1R-vaccinated: Developed severe clinical symptoms but recovered. 4pox-vaccinated: Clinically normal & survived. 3 animals (one negative control, one L1R-vaccinated & one 4pox-vaccinated) developed huge number of lesions at injection site.
Earl, Americo, Wyatt, Eller, Whitbeck, Cohen, Eisenberg, Hartmann, Jackson, Kulesh, Martinez, Miller, Mucker, Schamblin, Zwiers, Huggins, Jahrting and Moss [38]	Lesions	Dryvax alone: Lesions developed & size grown on days 7–10. MVA + Dryvax: Smaller & less indurated lesions. Lesions healed rapidly compared to Dryvax groups.
	Antibody responses	MVA + MVA & MVA + Dryvax: Detected at week 1, peaked between week 2–4 & boosted within one week post-booster vaccination. Antibody titres were 10× higher than single Dryvax vaccination. Single Dryvax: Antibody response peaked after 4 weeks.
	Neutralising antibodies	MVA + MVA & MVA + Dryvax: Detected after 1st vaccine & became higher after 2nd vaccine. However, there is not statistically significant difference between these groups.

**Table 7:** Characteristics of treatment combination tested in preclinical studies.

Ref.	Samples	Intervention	Comparison	Method
Wei, et al. [39]	Cynomolgus monkeys, *Macaca fascicularis* (n = 18 F, aged 3–6 years, weighed 2–4 kg)	Combination of 20 mg/kg of cidofovir and 2 ± 10^5^ PFU Dryvax vaccine.	Mock-vaccinated (Saline)	Randomly distributed into 3 groups of 6; (1) Control (Mock-vaccinated-Saline) (2) Dryvax vaccinated on day 0 (3) Dryvax vaccinated on day 0 + cidofovir IV. All animals were challenged with 5.0 × 10^7^ PFU of monkeypox virus strain Zaire 79, 55 days after immunization.

**Table 8:** Characteristics of treatment combination tested in preclinical studies (Outcome measures).

Ref.	Outcome Measures	Findings
Wei, Huang, Fortman, Wang, Shao and Chen [39]	Skin lesions & vaccinia viral loads.	Control group: Manifested skin rashes on day 4 & developed into a mean of 1000 lesions/animal on days 9 & 12 post-challenge. Dryvax vaccinated animals: All manifested rashes on injection site on day 3 post-vaccination then developed into blisters of ~120 mm^2^ on day 10. Rashes continued for 18 days before scabbing. All had viral mRNA detected at days 4 & 7. Dryvax + cidofovir-treated animals: No or small rashes of <20 mm^2^ (*p* < 0.01 compared to Dryvax-alone group). Rashes healed more rapidly. 3 animals had very low level of viral mRNA.
	Dryvax-elicited antibody and T-cell immune responses	Dryvax + cidofovir caused significant reduction in Dryvax elicited antibody responses. Mean titres of titre were one log less than Dryvax alone group.

**Table 9:** Characteristics of other potential therapeutic treatments tested in preclinical studies.

Ref.	Samples	Intervention	Comparison	Method	
Mucker, et al. [40]	Marmoset (male & female, weighing 233–437 g)	40 mg/kg of Human-chimeric monoclonal antibodies, c7D11 (human-murine chimeric antibodies) and c8A (human-chimpanzee chimeric antibodies).	PBS	Animals were administered with 20 mg/kg of each antibody 24 h via subcutaneous route before challenged with 50 μL of virus into each nare (total 100 μL). 2 control animals received PBS and a third were administered 40 mg/kg of human chimeric, non-poxvirus targeted monoclonal antibody (BioFactura).	
Johnston, et al. [41]	VA-9, VN36, and VA(R645) cells, monolayers of HeLa cells and normal human dermal fibroblast	0, 200, 400, 600, 800, 1000, 2000, 3000, 4000, or 5000 U/mL of **human IFNb 1a**	Placebo (untreated)	MPXV Inhibition in pre-treated, post-treated and primary human cells	MPXV inhibition by IFN-induced MxA
				Control group; monolayers of HeLa cells and normal human dermal fibroblast were either untreated or treated with varying doses of human IFNb 1a 24 h before challenge with MPXV-Zaire at an MOI of 5. Cells then harvested 24 h later, lysed by freeze-thawing/sonification and virus titres measured. Cells were observed using fluorescence microscopy. Post-treated; HeLa cells challenged with virus and treated with 2000 U/mL of IFNb at 0, 2, 4, 6, 8, or 12 h post-challenge.	VA-9, VN36 (control), and VA(R645) cells challenged with MPXV-Zaire or MPXV-GFP-tdTR (recombinant virus) at an MOI of 5. Cells then harvested 24 h later, lysed by freeze-thawing/sonification and virus titres measured. Cells were observed using fluorescence microscopy.

**Table 10:** Characteristics of other potential therapeutic treatments tested in preclinical studies (Outcome measures).

Ref.	Outcome Measures	Findings
Mucker, Wollen-Roberts, Kimmel, Shamblin, Sampey and Hooper [40]	Survival & clinical signs	Antibodies-treated animals did not manifest any clinical signs of disease whereas untreated animals experiences signs or death between day 13 to 18. All treated animals survived except animal #9 who did not exhibit any signs until day 24 when there was lack of appetite observed and a lesion was seen on its chin. The difference in survival rate is statistically significant when compared to untreated animals.
	vDNA	C7D11 was demonstrated to decrease the virus by more than 89% for >1250 PFU/mL, 96% for 210 PFU/mL and 100% for 55 PFU/mL.
	Immune cells	Level of WBC, lymphocytes, monocytes and granulocytes were seen to have increase in control animals. In treated animals, the monocyte levels fluctuated, and the platelet levels were in normal level.
Johnston, Lin, Connor, Ruthel, Goff and Hensley [41]	MPXV Inhibition in pre-treated, post-treated and human primary cells	In pre-treated HeLa cells, MPXV inhibition observed at 600 U/mL onwards. 2000 U/mL reduced MPXV by 91%. No observable toxicity shown in cells. GFP expressed by MPXV-GFP-tdTR were seen to decrease. In post-treated cells, virus was reduced by 91% when treated at 6–8 h post-challenge. In pre-treated human primary cells, virus was reduced by 95% with 25 U/mL of IFNb.
	MPXV inhibition by IFN-induced MxA in pre-treated cells	91% reduced observed in VA-9 cells.
